# *Staphylococcus aureus* ST398 Virulence Is Associated With Factors Carried on Prophage ϕSa3

**DOI:** 10.3389/fmicb.2019.02219

**Published:** 2019-09-24

**Authors:** Ayesha Kashif, Jo-Ann McClure, Sahreena Lakhundi, Michael Pham, Sidong Chen, John M. Conly, Kunyan Zhang

**Affiliations:** ^1^Centre for Antimicrobial Resistance, Alberta Health Services/Alberta Public Laboratories/University of Calgary, Calgary, AB, Canada; ^2^Department of Epidemiology and Health Statistics, Guangdong Pharmaceutical University, Guangzhou, China; ^3^Department of Pathology and Laboratory Medicine, University of Calgary, Calgary, AB, Canada; ^4^Department of Microbiology, Immunology and Infectious Diseases, University of Calgary, Calgary, AB, Canada; ^5^Department of Medicine, University of Calgary, Calgary, AB, Canada; ^6^The Calvin, Phoebe and Joan Snyder Institute for Chronic Diseases, University of Calgary, Calgary, AB, Canada

**Keywords:** *Staphylococcus aureus*, multilocus sequence type (ST), livestock-associated *Staphylococcus aureus* ST398, strain lineage, virulence, *Caenorhabditis elegans*, whole genome sequences (WGS), prophage ϕSa3

## Abstract

An increasing number of severe infections caused by *Staphylococcus aureus* ST398 strains has been observed. However, it has not been elucidated whether all ST398 strains are equally virulent. We collected 13 strains from China and Canada to test in a *Caenorhabditis elegans* infection model and compared their whole genome sequences (WGS) to explore potential insights into their virulence. All isolates belonged to ST398-methicillin-susceptible *S. aureus* (MSSA) with variant *spa* types (t034, t571, t1451, t1250). Pulsed field gel electrophoresis (PFGE) and WGS analyses showed that the 13 isolates clustered into 3 genomic types (Types A-C). WGS and prophage phylogenetic analyses also revealed that the strains could be divided into 3 phage groups (Groups 1–3), which correlated with high-, moderate-, and low-nematocidal activities, with mean killing rates of 94, 67, and 40%, respectively. Group 1 carried ϕSa3-Group 1 (ϕSa3-G1), Group 2 carried ϕSa3-G2, and Group 3 lacked ϕSa3. Interestingly, strain GD1706 (that genetically clustered within Type C) and strain GD487 (within Type B) both carried ϕSa3-G1 like phages and killed 92% of the nematodes, similar to the Type A strains carrying ϕSa3-G1. This study demonstrated that different ST398 sub-lineages possess variable virulence capacities, depending on the presence or absence, as well as the structure of the prophage ϕSa3 that carries virulence factors.

**IMPORTANCE:**

Since first being reported in the early 2000s, *Staphylococcus aureus* ST398 has not only become recognized as a frequent colonizing strain in economically important livestock animals, but has also proven to be a concern for infection in humans and, in particular, has been linked to higher rates of severe invasive human infections. We collected ST398 strains from China and Canada to test in a worm (*Caenorhabditis elegans*) infection model and compared their whole genome sequences to gain insight into pathogenesis. We have shown that different ST398 sub-strains differ in their virulence potential based on the presence or absence and structure of prophage ϕSa3, which carries important virulence factors. Our observations suggest that ST398 strains are relatively heterogeneous from a clinical perspective, and more studies are needed to differentiate between virulent and non-virulent ST398 strains to determine the true global spread of relevant sub-strains.

## Introduction

Since first being reported in the early 2000s in association with livestock (LA), multilocus sequence type ST398 *Staphylococcus aureus* has become recognized as a significant colonizing strain in livestock animals and occasionally acting as a pathogen causing infections in livestock, and companion animals, with the majority of strains displaying a multi-resistant phenotype and reported as methicillin resistant (MRSA) (McNamee and Smyth, 2000; Menzies and Ramanoon, 2001; Bradley, 2002; Voss et al., 2005; Springer et al., 2009; Cuny et al., 2010; van Duijkeren et al., 2010; Weese, 2010; Graveland et al., 2011; Dorado-Garcia et al., 2013). Human colonization and infection with LA-ST398-MRSA was initially reported among swine farmers in France and the Netherlands in the early 2000s, affecting people working in close contact with livestock and other farm animals, with the human isolates genetically linked to those collected from animals (Armand-Lefevre et al., 2005; Voss et al., 2005; Huijsdens et al., 2006; van Belkum et al., 2008; van Rijen et al., 2008; Grisold et al., 2010; Graveland et al., 2011). However, since these initial reports, ST398 strains have subsequently been reported as causing infections in humans in the absence of livestock exposure, with isolates predominantly described as being methicillin-sensitive (MSSA) (Uhlemann et al., 2012a, 2013; Brunel et al., 2014).

In humans, ST398 *S. aureus* infections can range from minor, localized disease to more severe invasive illnesses (Witte et al., 2007; van Belkum et al., 2008; Yu et al., 2008; Rasigade et al., 2010). There has been recognition of a number of severe infections in young healthy people caused by ST398-MSSA strains, most of which were acquired in the absence of animal contact (Rasigade et al., 2010; Valentin-Domelier et al., 2011; Brunel et al., 2014). ST398-MSSA has been reported as the etiological agent in cases of blood stream infection (Valentin-Domelier et al., 2011) and pneumonia (Rasigade et al., 2010), and has been isolated from patients in intensive care units (Brunel et al., 2014). These reports have sparked concerns that these MSSA strains represent a more virulent ST398 subtype, with an evolutionary tendency towards augmented pathogenicity specifically in humans. Consequently, it is important to identify if any genetic modifications have led to the appearance of these more virulent infections. While reports have investigated the increasing virulence of ST398 strains, they focus on genetic factors related to toxicity of the group as a whole (Yu et al., 2008; Uhlemann et al., 2012b; Bonesso et al., 2016). No reports have looked specifically to see if there are differences in virulence between sub-lineages within the ST398 MSSA strains. We therefore examined ST398-MSSA strains from China and Canada in order to test the strains in a *Caenorhabditis elegans* infection model, with the goal of determining if virulence differed between members within this group. Whole genome sequence (WGS) analysis of the strains was also done, with the aim of revealing insights into the virulence of this emerging pathogen.

## Materials and Methods

### Bacterial Strains

ST398 isolates were originally obtained from patients in the STI (sexually transmitted infection) clinic and the CUPS (Calgary Urban Project Society) clinic in Calgary, AB, Canada, in 2014, with samples being collected from nasal (N) or groin (G) swabs, as previously described (Ugarte Torres et al., 2017). In addition, colonization samples were obtained from public community school students or hospital patients during a MSSA/MRSA epidemiological prevalence survey during February-April 2010 in Guangzhou, Guangdong, China. The protocols were approved by the University of Calgary Conjoint Health Research Ethics under the Certification No.: REB13-0219, and the Ethics Committee of the First Affiliated Hospital/School of Clinical Medicine of Guangdong Pharmaceutical University under the Ethics No. 2011(1), respectively. Written informed consent was obtained from all participants.

The Canadian epidemic MRSA reference strains CMRSA1-10 were provided by the National Microbiology Laboratory, Health Canada, Winnipeg, Manitoba, Canada. The U.S. epidemic MRSA reference strains USA100-USA800 (NRS382, NRS383, NRS384, NRS123, NRS385, NRS22, NRS386, and NRS387, respectively), and the *C. elegans* control strain NCTC8325 were obtained through the Network on Antimicrobial Resistance in *Staphylococcus aureus* Program (NARSA). *C. elegans* control strain M92 was kindly provided by Dr. T. Louie from the University of Calgary, Canada.

### Strain Molecular Characterization

Staphylococcal ST398 isolates were fingerprinted by pulsed field gel electrophoresis (PFGE) after digestion with *Cfr*9I following a modified protocol (Mulvey et al., 2001; Bosch et al., 2010). PFGE-generated DNA fingerprints were digitized and analyzed with BioNumerics Ver. 6.6 (Applied Maths, Sint-Martens-Lattem, Belgium) using a tolerance of 1.5%. Isolates were tested for methicillin resistance using an in-house polymerase chain reaction (PCR) assay for the *mecA* gene, which was also used for detecting the PVL genes (Zhang et al., 2008). The isolates were further characterized with Staphylococcal protein A (*spa*) typing (Harmsen et al., 2003), multilocus sequence typing (MLST) (Enright et al., 2000), and accessory gene regulator (*agr*) typing (Peacock et al., 2002).

### DNA Sequencing and Whole Genome Sequence Analysis

Genomic DNA for each of the ST398 strains was isolated using phenol:chloroform extraction. All isolates were sequenced with Illumina MiSeq technology (2 × 250 bp), and representative strains from each group (GD705, 239G and GS1677) were further sequenced using Pacific Biosciences (PacBio) RSII sequencing technology (McGill University Génome Québec Innovation Centre) to facilitate genome assembly. Hybrid sequence assembly was performed using both read sets when available, or Illumina data was assembled using a reference guided assembly protocol (DNASTAR Lasergene v15.1, Madison, WI, United States). A further 4 strains (GD1108, GD1696, GD1706, and GD487) were sequenced with PacBio because of poor reference guided assembly results. The following genomes were available in the NCBI GenBank database; N315 (BA000018), RIVM1295 (CP013616), ISU926 (CP017091), E154 (CP013218), RIVM3897 (CP013621), 08BA012176 (CP003808), S0385 (AM990992), RIVM1607 (CP013619), and 08S00974 (CP020019).

Single nucleotide polymorphism (SNP) whole genome sequence (WGS) phylogenetic analysis was performed using CSI Phylogeny v1.4 with default settings, using strain N315 (BA000018) as the reference and rooting genome (Center for Genomic Epidemiology, Lyngby, Denmark). Phylogenetic trees were visualized with FigTree v1.4.3 (Institute of Evolutionary Biology, University of Edinburgh, Edinburgh, United Kingdom). Genetic relatedness was calculated using *in silico* DNA–DNA hybridization using the online software GGDC 2.1 with formula 3 (Meier-Kolthoff et al., 2013). Formula 3 was chosen due to the highly related nature of the isolates, all fully sequenced and sharing similar sized genomes. Blast ring images were generated using BRIG v0.95 (Alikhan et al., 2011). Prophage identification and annotation was conducted using PHASTER software (Zhou et al., 2011; Arndt et al., 2016), and the comparisons were done using Easyfig (Sullivan et al., 2011). Prophage phylogenetic analysis was also conducted using CSI Phylogeny v1.4 with default settings, using prophage phi 13 (ϕSa3) from strain NCTC 8325 (NC_004617) as reference. Mauve Alignment Distance Matrices (MADM) for the prophage phylogenetic distance were calculated with DNASTAR Lasergene Version 15.3.0.66 (DNASTAR, Inc., Madison, WI, United States). Virulence genes were identified with Virulence finder 1.5 (Joensen et al., 2014) and oriTfinder v1.0 (Li et al., 2018), using the assembled PacBio sequences when available, or with Illumina reads when it was not available.

### *C. elegans* Survival Experiments

Bristol N2 *C. elegans* nematodes were maintained on nematode growth medium plates spread with *Escherichia coli* strain OP50, at 25°C, as per established techniques (Stiernagle, 2006). *C. elegans* survival assays were performed as previously described (Wu et al., 2010). Briefly, 35 mm Tryptic soy agar plates were inoculated with test *S. aureus* strains and incubated at 37°C for 3 h. Approximately 30 nematodes were subsequently added to each plate and incubated at 25°C, with survival monitored every 24 h over a 5-day period. Killing rates were calibrated using the strains NCTC8325 and M92 as the virulent and the avirulent controls, respectively, as (% death_*Isolate*_ -% death_*M92*_)/(% death_8325_ -% death_*M92*_), with mean killing rates for individual strains and group related strains determined as the mean of 3–5 experimental replicates. *C. elegans* survival curves, comparisons of survival curves (calculated with Mantel-Cox test), and mean killing rates were generated using GraphPad Prism 7 (GraphPad Software, La Jolla, CA, United States). A *p*-value ≤ 0.05 was considered to be statistically significant.

## Results

### ST398-MSSA Strains Cluster Apart From Dominant MRSA Lineages and Subdivide Into 3 Genomic Types

**FIGURE 1:**
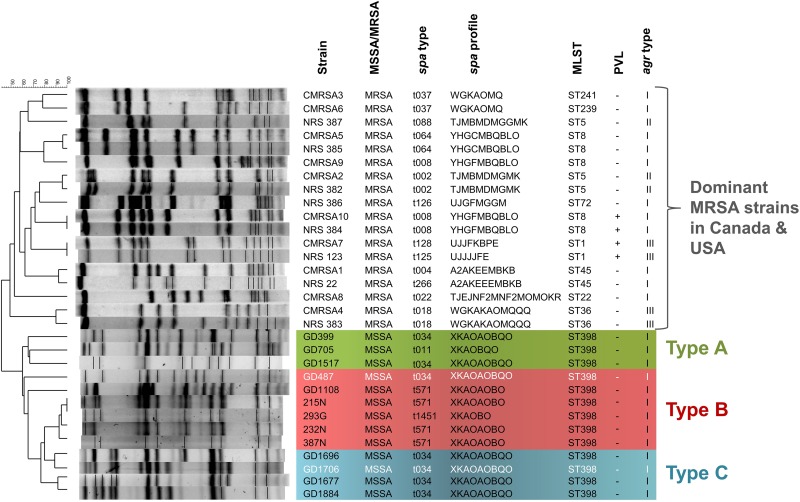
The ST398 MSSA isolates are distinct from other national and international MRSA lineages, and are subdivided into 3 genomic Types. Pulsotype and molecular characterization of the ST398 isolates and their relatedness to other Canadian (CMRSA1-10) and United States (USA1000-800) epidemic strains. Pulsotype generated following digestion with *Cfr*9I. Green shading denoting genomic Type A, red shading denoting genomic Type B, and blue shading denoting genomic Type C. Strains in white font belong to a different phage group than other members of the Type. *spa*, staphylococcal protein A; MLST, multilocus sequence typing; PVL, Panton-Valentine leukocidin; *agr*, accessory gene regulator.

In a total of 13 ST398-MSSA isolates collected from 2 geographical locations, 9 isolates (GD399, GD487, GD705, GD1108, GD1517, GD1677, GD1696, GD1706, GD1884) were obtained from school students or patients in Guangzhou, China, while 3 (215N, 232N, 293G) were obtained from the STI clinic and 1 (387N) was obtained from the CUPS clinic in Calgary, Canada. PFGE typing and molecular analysis revealed that all of our ST398 isolates clustered apart from other dominant MRSA strains in Canada and United States described to date, sharing only 40% similarity with these other strains (Figure 1). Strains GD399, GD705 and GD1517, all from China, appear to be separate from the other strains, forming a sub-group sharing 65–67% similarity of Dice coefficient of correlation (DCC). Similarly, strains 215N, 293G, 232N, and 387N, all from Calgary, are closely related and appear to belong to a sub-group, sharing 93–100% similarity. Strains GD1696 and GD1706, and GD1677 and GD1884 are distinct from the previously mentioned sub-groups, but are related to each other, sharing 77–92% similarity between them. Strains GD487 and GD1108 did not appear to be closely related to any of the sub-groups by PFGE analysis. All 13 isolates were determined to be methicillin sensitive, PVL negative and *agr* type I. GD399, GD1517, GD487, GD1696, GD1706, GD1677 and GD1884 all belonged to *spa* type t034 (XKAOAOBQO), while GD705 was type t011 (XKAOBQO), GD1108, 215N, 232N and 387N carried *spa* type t571, and 293G carried *spa* type t1451 (XKAOBO) (Figure 1).

**FIGURE 2:**
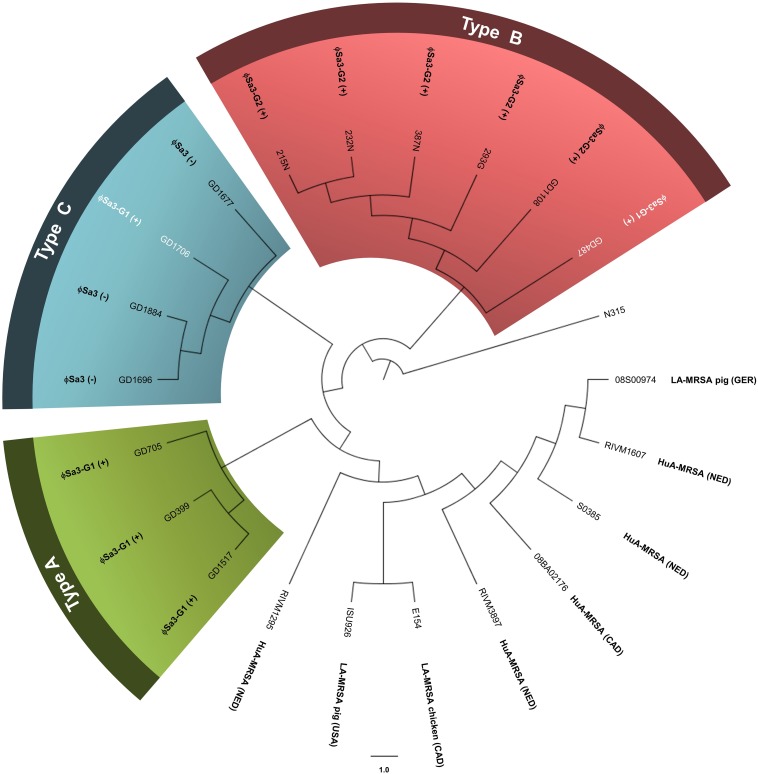
Phylogenetic analysis of genome-wide SNPs confirms 3 genetic sub-lineages, distinct from other reference ST398 strains. The cladogram (circular representation) comparing our ST398 isolates with human and animal sourced ST398 from around the world indicates that three genomic Types (A, B, and C) were present, and separate from other global ST398. The presence or absence of ϕSa3 is noted for each strain. Green shading denoting Type A, red shading denoting Type B, and blue shading denoting Type C. Strains in white font belong to a different phage group than other members of the Type. N315 (BA000018), the reference genome, was used as the out-group to root the tree. The branch lengths are arbitrary. Global strains and accession numbers are as follows: RIVM1295 (CP013616), ISU926 (CP017091), E154 (CP013218), RIVM3897 (CP013621), 08BA012176 (CP003808), S0385 (AM990992), RIVM1607 (CP013619), and 08S00974 (CP020019). Strain sources are indicated where known and applicable. MRSA, methicillin-resistant Staphylococcus aureus; HuA, human-associated (isolated from a human); LA, Livestock-associated (isolated from an animal); Ger, Germany; NED, Netherlands; CAD, Canada. (+) strain carries the phage; (–) strain does not carry the phage.

WGS analysis of the 13 ST398 strains revealed that they clustered apart from previously reported ST398 strains, and into 3 genomic types. The genomes of all the ST398 isolates were sequenced and found to be similar in size, ranging from 2.72 to 2.88 Mb, with GD1108 and GD1706 having plasmids. Phylogenetic analysis of genome-wide SNPs and *in silico* DNA–DNA hybridization (*is*DDH) analysis were used to determine the genetic relationship of the strains and estimate their genome-genome distances. SNP phylogenetic analysis of the 13 isolates, along with representative global ST398 strains with whole genome sequences available in GenBank, determined that our ST398 clustered into 3 genomic types (Types A, B, and C), distinct from the other international ST398 strains (Figure 2). The average genome to genome distance between our 13 strains and the international ST398 strains was 98.4%, with an average *is*DDH of 91.6% with respect to the outgroup strain, N315. Genetic distances between the 3 types were closer, showing that, while they are distinct, they still represent a subset of the closely related ST398 lineage. Types A and B had an average *is*DDH of 99.54%, whereas Type B and C had an average of 98.51%, and Types A and C an average *is*DDH of 98.6%.

### ST398-MSSA Strains Showed Differential Levels of Virulence in a *C. elegans* Infection Model

**FIGURE 3:**
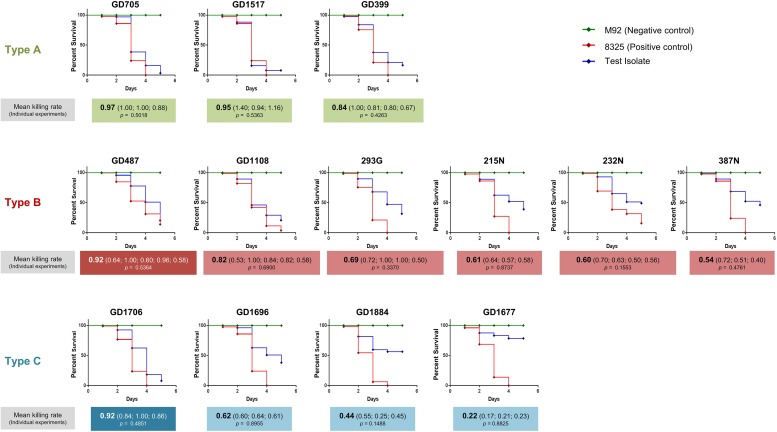
*Caenorhabditis elegans* survival assays showed differential virulence potentials for the ST398 isolates. *C. elegans* survival curves indicate that the strains possessed a range of toxicities, including high, moderate and low levels. Highly toxic control strain 8325 is indicated with a red line, non-toxic control strain M92 is indicated with a green line, and the ST398 isolates are indicated with a blue line. Strains in white font belong to a different phage group than other members of the Type. Mean killing rates for each strain are indicated below the curve, along with killing rates for each replicate. *P*-values for all replicates are included, with *p* < 0.05 considered significant.

Having determined that the ST398-MSSA isolates separated into 3 genetically related types, a *C. elegans* infection model was used to assess the virulence of each type. Mean killing rates for each strain, survival curves, and statistical significance are shown in Figure 3. Members of Type A had mean killing rates ranging from 0.97 for GD705 (Reproducible, *p* = 0.5018), to 0.95 for GD1517 (Reproducible, *p* = 0.5363), and 0.84 for GD399 (Reproducible, *p* = 0.4263) (Figure 3). Members of Type B had mean nematode killing rates ranging from 0.82 for GD1108 (Reproducible, *p* = 0.6900), to 0.69 for 239G (Reproducible, *p* = 0.3370), 0.61 for 215N (Reproducible, *p* = 0.8737), 0.60 for 232N (Reproducible, *p* = 0.1553), and 0.54 for 387N (Reproducible, *p* = 0.4761). GD487 was particularly high, with a mean killing rate of 0.92 (Reproducible, *p* = 0.5364) (Figure 3). Members of Type C had mean killing rates ranging from 0.62 for GD1696 (Reproducible, *p* = 0.8955), to 0.44 for GD1884 (Reproducible, *p* = 0.1488) and 0.22 for GD1677 (Reproducible, *p* = 0.8825). GD1706 was unusually high, with a mean killing rate of 0.92 (Reproducible, *p* = 0.4851) (Figure 3). It was noted that, despite sharing highly similar genetic backgrounds, there were still variations in virulence between members of a genomic Type. Strains in Type A were more uniform and all trended towards higher toxicity. The majority of strains in Type B trended towards moderate toxicity, however, as mentioned, GD487 displayed unusually high levels of toxicity. Similarly, the majority of strains in Type C trended towards lower toxicity, with GD1706 showing unusually high levels of toxicity. These observations suggested that virulence was not related directly to the genomic background, but rather that some other factor played a role.

### Prophages in the Strains

**FIGURE 4:**
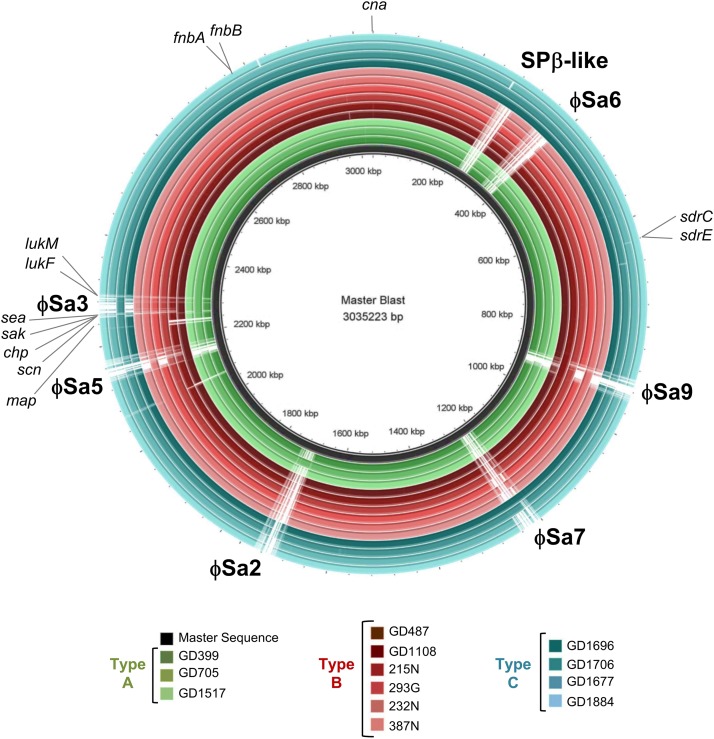
Six prophages and multiple virulence genes are located within the ST398 genomes, with their presence varying between the strains. BRIG alignment of the ST398 strains, with the locations of each phage and important virulence gene marked. Rings from inner to outer are indicated in the legend and the groups color as green for genomic Type A, red for Type B, and blue for Type C. A master-sequence was created incorporating all of the relevant phages and virulence genes and used as the reference sequence for generating the BRIG alignment. *cna*, collagen adhesin precursor; *sdrC*/*E*, Ser-Asp rich fibrinogen-binding bone sialoprotein-binding protein C and E; *map*, extracellular proteins Map; *scn*, staphylococcus complement inhibitor; *chp*, chemotaxis inhibitory protein; *sak*, staphylokinase; *sea*, staphylococcus enterotoxin A; *lukF*, leukotoxin F subunit; *lukM*, leukotoxin M subunit; *fnbA*/*B*. fibronectin-binding protein A and B.

A BLAST ring comparison of all the ST398-MSSA isolates indicated that multiple prophages were present in their genomes, with carriage of some prophages being type specific (Figure 4). All members of Type A (represented by green rings) carried ϕSa3, with some structural variations, and one member (GD705) also carried ϕSa2. Similar to Type A, all members of Type B (represented by red rings) carried ϕSa3, while 1 strain (GD487) carried ϕSa5, and 3 strains (GD1108, 232N and 215N) had ϕSa9. All members of Type C (represented by blue rings) carried ϕSa6, but only 1 (GD1706) carried both ϕSa3 andϕSa9, 1 (GD1696) carried ϕSa7, and 3 (GD1706, GD1696, and GD1884) carried the SPβ-like element (phage carriage summarized in Table 1). While carriage of the other prophages appears to be sporadic, ϕSa3 was found in all of the strains with high and moderate nematocidal activity, which prompted a deeper investigation into that phage.

**TABLE 1:** Genetic factors differing among the ST398 strains.

					**ϕSa3 related virulence genes**											**Other phages**						**Other virulence genes**					
							
**Phage Group**	**Strain**	**Genomic Type**	**Mean killing rate**	**ϕSa3**	***attL***	***scn***	***chp***	***sak***	***sea***	***attR***	***lukF***	***lukM***	**Lysogeny module**	**Replication module**	**Transcription module**	**SPβ-like**	**ϕSa6**	**ϕSa9**	**ϕSa7**	**ϕSa2**	**ϕSa5**	***sdrC***	***sdrE***	***map***	***fnbA***	***fnbB***	***cna***
Group 1 (Higher Virulence)	GD705	A	0.97	**+**	✓	**+**	**+**	**+**	**+**	✓	**+**	**+**	ant4b	dnaD1a	dut3	–	–	–	–	**+**	–	**+**	**+**	–	**+**	**+**	**+**
	GD1517	A	0.95	**+**	✓	**+**	–	–	–	✓	**+**	**+**	ant4a	dnaD1a	dut2	–	–	–	–	–	–	**+**	–	**+**	**+**	–	**+**
	GD399	A	0.84	**+**	✓	**+**	–	–	–	✓	**+**	**+**	ant4a	dnaD1a	dut2	–	–	–	–	–	–	**+**	**+**	**+**	**+**	**+**	**+**
	GD487	B	0.92	**+**	✓	**+**	–	**+**	**+**	✓	–	–	ant4b	dnaD1b	dut3	–	–	–	–	–	**+**	**+**	**+**	**+**	**+**	**+**	**+**
	GD1706	C	0.92	**+**	✓	**+**	**+**	–	–	✓	–	–	ant1a	dnaD1a	dut3	**+**	**+**	–	–	–	–	**+**	**+**	**+**	**+**	**+**	**+**

Group 2 (Moderate Virulence)	GD1108	B	0.82	**+**	✓	**+**	**+**	–	–	✓	**+**	**+**	ant4b	dnaD1b	dut1	–	–	**+**	–	–	–	**+**	**+**	**+**	**+**	**+**	**+**
	293G	B	0.69	**+**	✓	**+**	**+**	–	–	✓	**+**	**+**	ant4b	dnaD1b	dut1	–	–	–	–	–	–	**+**	**+**	**+**	**+**	**+**	**+**
	215N	B	0.61	**+**	✓	**+**	**+**	–	–	✓	**+**	**+**	ant4b	dnaD1b	dut1	–	–	**+**	–	–	–	–	–	**+**	**+**	**+**	–
	232N	B	0.60	**+**	✓	**+**	**+**	–	–	✓	**+**	**+**	ant4b	dnaD1b	dut1	–	–	**+**	–	–	–	**+**	**+**	**+**	**+**	**+**	**+**
	387N	B	0.54	**+**	✓	**+**	**+**	–	–	✓	**+**	**+**	ant4b	dnaD1b	dut1	–	–	–	–	–	–	–	**+**	**+**	–	**+**	–

Group 3 (Lower Virulence)	GD1696	C	0.62	–	n/a	n/a	n/a	n/a	n/a	n/a	n/a	n/a	n/a	n/a	n/a	**+**	**+**	–	**+**	–	–	**+**	**+**	**+**	**+**	**+**	**+**
	GD1884	C	0.44	–	n/a	n/a	n/a	n/a	n/a	n/a	n/a	n/a	n/a	n/a	n/a	**+**	**+**	–	–	–	–	**+**	**+**	**+**	**+**	**+**	**+**
	GD1677	C	0.22	–	n/a	n/a	n/a	n/a	n/a	n/a	n/a	n/a	n/a	n/a	n/a	–	**+**	–	–	–	–	**+**	**+**	**+**	**+**	**+**	**+**

*Note: The presence of ϕSa3 related genes, other phages, and other chromosomally encoded virulence genes/markers are noted, with gene order in the table corresponding to gene order on the chromosome. n/a, not applicable; (**+**), phage or gene present; (–), phage or gene absent; (✓), attachment site present at relative location. *att*L, left attachment site; *sak*, staphylokinase; *chp*, chemotaxis inhibitory protein; *scn*, *Staphylococcus* complement inhibitor; *sea*, *Staphylococcus* enterotoxin A; *att*R, right attachment site; *lukF*, leukotoxin F subunit; *lukM*, leukotoxin M subunit. *sdrC*/*E*, Ser-Asp rich fibrinogen-binding bone sialoprotein-binding protein C and E; *map*, extracellular proteins Map; *fnbA*/*B*. fibronectin-binding protein A and B; *cna*, collagen adhesin precursor. All strains were negative for the livestock associated marker *tetM*.*

### ϕSa3 and Its Structure Is Associated With ST398-MSSA Strain Virulence

**FIGURE 5:**
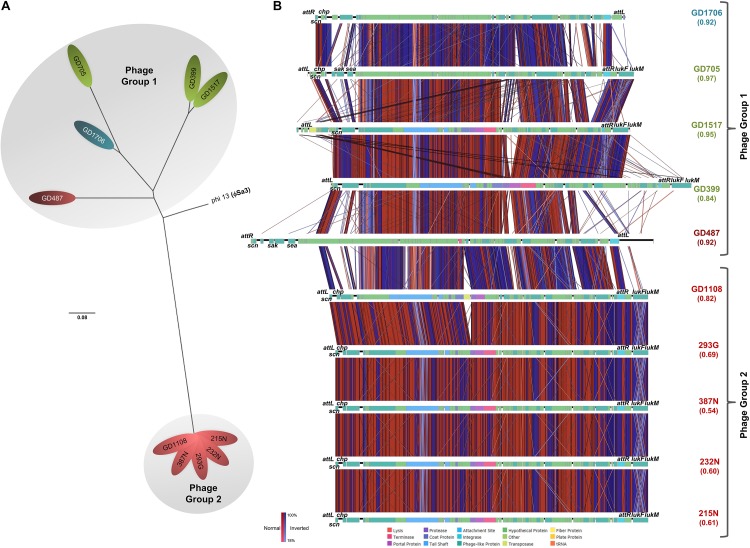
ϕSa3 comparison among the ST398. **(A)** Unrooted SNP phylogeny (radial representation) of ϕSa3 shows that the phages present in the 3 genomic Types belonged to two phage groups, 1 and 2. The Group 2 phages are highly related, and distinct from Group 1 phages. The phage present in GD487 (from genomic Type B) and GD1706 (from genomic Type C) are more similar to the phages present in Type A strains and, together, form phage Group 1. This is an unrooted tree with phi 13 (ϕSa3) from strain NCTC 8325 (NC_004617) as reference and the scale bar indicates number of substitutions per site. Green shading denoting Type A, red shading denoting Type B, and blue shading denoting Type C. Strains in white font belong to a different phage group than other members of the Type. **(B)** EasyFig comparisons of all the ϕSa3 shows that ϕSa3-G1 in Group 1 are related, but still contain variability, while ϕSa3-G2 in Group 2 are very similar. Virulence genes and attachment sites are indicated. *att*L, left attachment site; *sak*, staphylokinase; *chp*, chemotaxis inhibitory protein; *scn*, staphylococcus complement inhibitor; *sea*, staphylococcus enterotoxin A; *att*R, right attachment site; *lukF*, leukotoxin F subunit; *lukM*, leukotoxin M subunit. Strains with Green font belong to genomic Type A, red font to Type B, and blue font to Type C.

SNP phylogenetic analysis of the phages, and the resulting dendrogram, indicated that the ϕSa3 phages present in Type B strains (GD1108, 387N, 293G, 232N, and 215N) were very closely clustered together with a mean MADM of 0.0001 (range: 0.0000-0.0001). Interestingly, the ϕSa3 phages present in the strains GD487 (Type B) and GD1706 (Type C) – with unusually high virulence patterns, were shown to be more closely related to the phage ϕSa3 from the Type A strains, forming a group with a mean MADM of 0.0250 (range: 0.0002–0.0391) (Figure 5A). Although still variant, the members within this group were more distinct from all members from the former group, with a mean MADM of 0.0506 (range: 0.0486–0.0537). As such, the phages were divided into three phage groups based on their phage phylogenetic similarity, ϕSa3-Group 1 (ϕSa3-G1), ϕSa3-Group 2 (ϕSa3-G2) as indicated in Figure 5A, with Group 3 representing strains lacking prophage ϕSa3. A closer examination of ϕSa3 revealed structural variations between the groups, however, strains with similar virulence possessed phages with similar composition (Figure 5B). ϕSa3 contains several genes linked to virulence, including the genes for *Staphylococcus* complement inhibitor (*scn*), chemotaxis inhibitory protein (*chp*), staphylokinase (*sak*), enterotoxin A (*sea*), leukotoxin-F subunit (*lukF*), and leukotoxin-M subunit (*lukM*). Structural alignments of all phages are shown in Figure 5B, with a summary of the phage related virulence genes noted in Table 1.

Phage Group 1 contained all 3 members of genomic Type A (GD705, GD1517 and GD399), 1 member of genomic Type B (GD487), and the only member of genomic Type C possessing ϕSa3 (GD1706). The Type A strains all contained ϕSa3-G1 inserted into the *hlb* gene at approximately 1,950 Kbp on their chromosomes. While the overall gene content in these phages varied significantly, all 3 strains had the *scn* genes in their ϕSa3-G1, with *chp*, *sak* and *sea*, also present in GD705 (see Figure 5B, summary in Table 1 and extended gene list in Supplementary Table 1). Additionally, all 3 strains contained the *lukF* and *lukM* genes external to the phage attachment sites. A detailed analysis of the phage functional modules revealed that both GD1517 and GD399 carried lysogeny modules with anti-repressor type ant4a, while GD705 had type ant4b. Similarly, GD1517 and GD399 had a regulation of transcription module with a dUTPase type dut2, while GD705 had type dut3. All 3 strains had type dnaD1a replication modules. Strain GD487 (Type B) had its ϕSa3 inserted in the reverse orientation into a different region of the chromosome; namely into an alpha/beta hydrolase near 1,238 Kbp, with the *hlb* gene at 2,006 Kbp remaining intact. ϕSa3-G1 of GD487 carried the *sak*, *scn* and *sea* genes, but lacked *lukF* and *lukM.* The phage in GD487 had an identical integrase as found in GD1517 and GD399 (Supplementary Table 1), lysogeny module ant4b, replication module dnaD1b, and the same regulation of transcription module as the Type A strains, dut3. Similar to GD487, strain GD1706 (Type C), also had ϕSa3 found in an atypical location and inserted in the reverse orientation. It was inserted into the cytochrome d ubiquinol oxidase subunit II gene at 1,172 Kbp, with an intact *hlb* gene found at 2,136 Kbp. The phage contained the *scn*, and *chp* genes, but lacked the *lukF* and *lukM* genes (Figure 5B and Table 1), and carried the identical integrase as found in ϕSa3-G1 of GD1517 and GD399 (Supplementary Table 1). In terms of the functional modules, GD1706 had the ant1a lysogeny module, dut3 regulation of transcription module, and dnaD1a replication module. Strains in phage Group 1 trended to have higher virulence levels (mean killing rate of 0.84–0.97), with a Group mean of 0.94.

Strains in phage Group 2 carried ϕSa3-G2 inserted into the *hlb* gene at approximately 1,950 Kbp on the chromosome. ϕSa3-G2 in strains 293G, 215N, 232N, and 387N were nearly identical in composition, each carrying *scn* and *chp* in the phage and *lukF* and *lukM* external to the phage attachment sites. Their phages differed by a few hypothetical proteins (≤6% of the genes) (structure and content summary in Figure 5B and Table 1, and detailed gene list in Supplementary Table 2). ϕSa3-G2 of GD1108 differed from the other 4 strains in that it contained a putative transposase mid-phage but was nearly identical to the rest throughout the remainder of the phage (Supplementary Table 2). Looking at the phage functional modules, strains GD1108, 293G, 387N, 232N, and 215N all had the same lysogeny module (ant4b), regulation of transcription module (dut1) and replication module (dnaD1b). The Calgary strains (293G, 232N, 387N and 215N) had similar phages with similar genomic modules and comparable levels of nematocidal toxicity (mean killing rates of 0.54–0.69), while GD1108, with the extra transposase had a higher level of toxicity (mean killing rate of 0.82) than the rest of the Group 2 members. The group as a whole, however, trended towards moderate toxicity, with a Group mean killing rate of 0.67.

Strains in phage Group 3 all belonged to genomic Type C, lacked ϕSa3, and had undisturbed *hlb* genes. Strains in this Group tended to have lower levels of nematocidal toxicity (mean killing rates of 0.22–0.62), with a group mean of 0.40.

Despite variances in nematocidal activity between strains within a phage Group, a comparison of the group average survival curves revealed that there was still a significant difference between the groups; Groups 1 and 2 (*p* = 0.0001), Groups 2 and 3 (*p* = 0.0006), and Groups 1 and 3 (*p* < 0.0001).

### Other Chromosomally Encoded Virulence Related Genes/Markers in ST398-MSSA

Other mobile genetic elements, including ϕSa2, ϕSa5, ϕSa6, ϕSa7, ϕSa9, and the SPβ-like element were sporadically carried by members of these ST398-MSSA strains, however, no known virulence genes were found in them, and no patterns were noted with respect to element presence and virulence. Strains GD1108 and GD1706 also carried plasmids, but no virulence genes were found on the GD1706 plasmid, and the only significant genes on the GD1108 plasmid were ones related to arsenic and cadmium resistance.

Looking further for genetic differences which could account for the varied toxicities seen between the strains, virulence genes elsewhere in the chromosome were compared. Using the online software *ori*T finder and virulence gene finder, 63 genes were examined. The majority of the genes showed no variability between the groups, however, the genes for Ser-Asp rich fibrinogen-binding bone sialoprotein-binding proteins (*sdrC*, *sdrE*), the MHC class II analog protein (*map*), fibronectin-binding protein (*fnbA, fnbB*) and collagen adhesin precursor (*cna*) showed minor variation in carriage between the strains. There were, however, no patterns which could be correlated to any specific group or virulence pattern seen (as shown in Table 1). Additionally, presence of the tetracycline resistance gene, *tetM*, associated with ST398 LA-MRSA was assessed. All strains examined were found to be negative for the *tetM* gene.

## Discussion

The whole-genome sequence analysis of a worldwide diverse collection of CC398 strains by Price et al. (2012) provided evidence that human-associated MSSA ST398 was the basal clade from which LA-MRSA ST398 had emerged. The basal MSSA ST398 is genetically different from LA-MRSA ST398 and usually carries phages (often ϕSa3) harboring the human virulence genes (such as *sak*, *chp*, and *scn*) that are not typically found in LA-MRSA ST398, and lacks the livestock-associated marker *tetM* (Price et al., 2012). These MSSA ST398 strains are usually community-associated (CA-MSSA ST398) and epidemiologically common in some Asian countries, and often cause more severe invasive diseases (Valentin-Domelier et al., 2011; van der Mee-Marquet et al., 2011; Price et al., 2012; Uhlemann et al., 2012b; Brunel et al., 2014; He et al., 2018). However, research into the specific factors that contribute to its virulence is limited to the group as a whole, with no reports investigating the virulence differences that may exist between members within the group. To that end, we examined the genetic background and virulence of a collection of ST398-MSSA strains isolated from mainland China and Calgary, Canada, and found that there are in fact differences in virulence noted between subgroups of the strain, and that carriage/structure of ϕSa3 appears to play a major role in that virulence.

Initial molecular analysis by PFGE indicated that all 13 of our isolates were distinct from Canadian and internationally dominant MRSA strains. Similarly, the cladogram based on phylogenetic analysis of genome-wide SNPs revealed that our ST398 isolates were related, but distinct from other global ST398 strains, and could be divided into 3 sub-lineages or genomic types. As a whole, our strains had a much greater genetic distance from other global ST398 strains, with an average isDDH 98.4%. Uhlemann et al. (2012b) previously noted that human associated (HuA) ST398 carry fewer mobile genetic elements (MGEs) as compared to livestock associated LA-ST398, which would contribute to the genetic separation from the pig and chicken isolates in the SNP phylogenetic tree. Our ST398 strains are all methicillin-sensitive and human-associated, lack the livestock-associated marker *tetM*, and most of them (except three isolates in the Type C, but still phylogenetically clustered together as compared to the other global ST398 strains) carries prophages ϕSa3 encoding the *sak*, *chp*, and *scn* human evasion genes. Taken together, these data support the notion that our HuA-ST398-MSSA strains could represent a distinct lineage of ST398 belonging to CA-MSSA ST398 category (Price et al., 2012; He et al., 2018).

The fact that our ST398-MSSA isolates, though closely related, still formed genetically distinct sub-lineages prompted us to investigate if they differed in their virulence and pathogenic properties. McClure and coworkers recently showed that a genetically closely related MRSA ST239 sub-lineage differed remarkably in its virulence and pathogenicity despite striking similarities in the genomes and antibiotic resistance profiles (McClure et al., 2018). Small changes such as the presence or absence of MGEs, or the disruption of genes, were postulated to account for the significantly different clinical presentations of the strains. To our knowledge, no report to date has described the different virulence potential of ST398 strains. We used a *C. elegans* infection model to assess the virulence of different ST398 strains and compared their WGS to gain potential insights into their pathogenic properties. Our results indicated that the strains not only had statistically significant differences in levels of virulence (low, moderate or high nematocidal activity), but WGS analysis showed the virulence corresponded to the phage groupings.

Of particular interest is the fact that strain GD1706, whose genetic background belonged to Type C, was found to be highly toxic and carried ϕSa3-G1, while other members of Type C trended towards lower toxicity and were devoid of phage. The presence of prophage ϕSa3 in all high and moderately virulent strains of Groups 1 and 2, and its absence from the low virulent strain of Group 3, suggests that ϕSa3 might play a crucial role in the virulence and pathogenicity of these ST398 strains. Uhlemann and co-workers previously described the importance of ϕSa3 in ST398 noting that, in their HuA-ST398, prophage ϕSa3 carried the human-specific immune evasion cluster (IEC) genes *chp* (chemotaxis inhibitory protein; CHIPS) and *scn* (staphylococcal complement inhibitor; SCIN) (Uhlemann et al., 2012b). The presence of these human virulence genes on our ϕSa3 supports the notion that the phage contributes to human host-specific adaptation of the strain, thereby contributing to its virulence.

A closer examination of ϕSa3 in our strains showed that, while all isolates in Groups 1 and 2 carried the phage, variations existed in their gene content, which could account for the virulence differences observed in the *C. elegans* infection model. This observation has previously been described in a study examining MGE in *S. aureus* ST239, where not only the presence or absence of MGE were postulated to play a role in determining the virulence of the strain, but also the gene content and structural properties of the MGEs (McClure et al., 2018). In the study, the structure and content of ϕSa3 differed between the strains, with the most virulent member carrying the full complement of genes (*sak*, *sea*, *scn*, and *chp*), and virulence of the other strains decreased proportionally as the genes were absent or mutated. Likewise, amongst our isolates, GD705 (from Type A, carrying ϕSa3-G1 belonging to Group 1) had the highest nematocidal activity (97%), and carried all 4 IEC genes (*scn*, *chp*, *sak* and *sea*). GD487 (from Type B but carrying ϕSa3-G1 belonging to Group 1) also had a higher number of IEC genes, with *sak*, *scn* and *sea* detected, and had a correspondingly higher level of virulence (92%) than the other Type B members (from Type B, carrying ϕSa3-G2 belonging to Group 2).

ϕSa3 functional modules may also play a role in determining strain virulence. *S. aureus* siphoviruses are divided into functional modules, including lysogeny, DNA replication, regulation of transcription, packaging, head and tail, tail appendices and lysis modules, of which a single gene has been shown to effectively define the types (Iandolo et al., 2002; Kahánková et al., 2010). A previous study by van Alen et al. (2018) characterized the phage modules in ST398 isolates, finding that there were variations present in each module within the lineage. In our study we saw similar module types to those reported by van Alen et al. (2018), with differences relatable to strain virulence. The lysogeny module in all our moderate virulence strains belonged to type ant4b, while the higher virulence strains belonged to ant1a or ant4a, but also contained ant4b. The lysogeny module consists of an integrase and excisionase gene, along with a cI-like repressor and anti-repressor, and is involved in the decision between the temperate/lysogenic life style (Kaiser, 1957; Ptashne and Hopkins, 1968; Simatake and Rosenberg, 1981; Lucchini et al., 1999). Similar to the lysogeny module, strains with moderate virulence had replication modules of type dnaD1b, while higher virulence strains carried type dnaD1a (with the exception of GD487 which carried type dnaD1b). Finally, the regulation of transcription module differed between the ST398 strain groups, with the moderately virulent strains carrying a dut1 type module and the high virulence strains carrying a dut2 or dut3 type of module. The dut1 is found in the majority of organisms, while dut2 and dut3 are members of the dUTPase_2 superfamily (Kahánková et al., 2010). How the differences in transcription module type may affect virulence is yet to be determined. Perhaps, as others have described (Sumby and Waldor, 2003; Goerke et al., 2006a, b; Weigel and Seitz, 2006; Kahánková et al., 2010; Tormo-Más et al., 2010, 2013; Ferrer et al., 2011; Penadés and Christie, 2015), expression of virulence genes within the phages and pathogenicity islands are upregulated upon induction, and the differing module types differ in their ability to induce or be induced. Regardless of the direct cause, it is intriguing that the modules tended to separate in a manner that could be related to the virulence groups. It is unlikely that any one factor is solely responsible for the varied toxicities observed, but rather that they function together to determine each strain’s level of virulence. And, despite the strong correlation between virulence and ϕSa3, other chromosomal components may also play a role and cannot be discounted. In our current study, the isolate GD1108 from the phage Group 2 (carrying prophage ϕSa3-G2 and tending to be moderate virulence) had a higher nematode killing rate (0.82) than the remaining members of Group 2, which was near to the low virulence range (0.84–0.97) from Group 1 (carrying prophage ϕSa3-G1 and tending to be high virulence). The same is true for the isolate GD1696 from the phage Group 3 (lacking prophage ϕSa3 and tending to be low virulence), which had a 0.62 nematode killing rate that overlapped with Group 2 virulence range (carrying prophage ϕSa3-G2 and tending to be moderate virulence). Although GD1108 had an extra transposase in its ϕSa3-G2, along with having an additional prophage (ϕSa9) and a plasmid, and likewise, GD1696 carried prophage ϕSa7, which the other members of Group 3 did not (Table 1 and Supplementary Table 2), the detailed mechanisms corresponding to their combinational effects on the nematocidal activity still remain to be elucidated.

To date, there is no human data to directly support the conclusions that different ST398 lineages possess differing levels of virulence. However, past studies have indicated that *C. elegans* is a robust host model to study the virulence and pathogenesis of bacteria (Tan et al., 1999a, b; Aballay et al., 2000; Couillault and Ewbank, 2002; Kurz et al., 2003). Wu and co-workers have shown that the nematocidal activity of MRSA correlates well with its isolation from clinically invasive anatomic sites vs. colonization site, concluding that *C. elegans* is a useful model to study the pathogenesis of MRSA (Wu et al., 2010). The group went on to further perform double-blinded virulence tests with the *C. elegans* host model, using isolates from an invasive outbreak strain (virulent), a non-invasive outbreak strain (intermediate virulent) and a colonization strain (avirulent) (Wu et al., 2012). As before, there was a high degree of correlation between the nematocidal rates noted and the invasiveness of the strain in humans. Together, these results strongly support the use of *C. elegans* as a host model to study the virulence of *S. aureus*, validating our choice to use it as a model to study the pathogenesis of ST398. Further studies will of course be needed to confirm if the different lineages of ST398-MSSA have different toxicities in humans, and if the presence and structure of ϕSa3 correlates with that.

## Conclusion

Our findings demonstrated the differences in virulence between members of a closely related ST398-MSSA lineage, using both the *C. elegans* virulence model as well as WGS analysis. We have shown that different ST398 sub-lineages differ in their virulence potential, correlating well with the presence or absence, as well as the structure of, prophage ϕSa3. Our observations suggest that ST398 strains may be relatively heterogenous from a clinical perspective, and more studies are needed to differentiate between virulent and non-virulent ST398 strains to determine the true global spread of the relevant sub-lineages.

## Data Availability Statement

The chromosomal genome sequence data have been deposited at GenBank under the accession numbers CP019593 (GD705), CP040229 (GD487), CP040230 (GD1108), CP019591 (293G), CP040232 (GD1706), CP040233 (GD1696) and CP019595 (GD1677), and SRA accession numbers SRX5802346 (GD1517), SRX5802629 (GD399), SRX5802683 (215N), SRX5802701 (232N), SRX5807140 (387N), and SRX5807290 (GD1884).

## Ethics Statement

The ethics protocols were approved by the University of Calgary Conjoint Health Research Ethics under the Certification No. REB13-0219 and the Ethics Committee of the First Affiliated Hospital/School of Clinical Medicine of Guangdong Pharmaceutical University under the Ethics No. 2011(1), respectively. Written informed consent was obtained from all participants.

## Author Contributions

KZ conceived, designed, and supervised the work. AK, J-AM, and MP performed the experiments and analyzed the data. SC and JC collected and provided the clinical isolates. J-AM, SL, and KZ structured and drafted the manuscript. J-AM, JC, and KZ reviewed and edited the manuscript.

## Conflict of Interest

The authors declare that the research was conducted in the absence of any commercial or financial relationships that could be construed as a potential conflict of interest.
