# Supplementary material for: Staphylococcus aureus ST398 Virulence Is Associated With Factors Carried on Prophage ϕSa3
Source: Front Microbiol. 2019 Sep 24;10:2219. doi: 10.3389/fmicb.2019.02219 (PMC6771273; doi:10.3389/fmicb.2019.02219)
Supplement: Supplementary file 1 [file Table_1.DOCX]

**Supplementary Table 1. Comparison of the *attL*/*attR* sites and the genes coding for proteins /hypothetical proteins among phage Group 1 (φSa3-G1) strains**

| ***attL*/*attR* sites and genes coding for proteins/hypothetical proteins** | **Strains** | | | | |
| --- | --- | --- | --- | --- | --- |
|  | **GD 705** | **GD 1517** | **GD 399** | **GD 1706** | **GD487** |
| **attL (TGTATCCGAATTGG)** | + | - | - | - | - |
| **attL (TGTATCCAGCAGAGG)** | - | + | - | - | - |
| **attL (TGTATCCAAACTGG)** | - | - | + | - | - |
| **attR (AGTCTCCAGTTTGG)** | - | - | - | + | - |
| **attR (TGTATCCATTATGG)** | - | - | - | - | + |
| hypothetical protein (gi526003548) | + | - | - | - | - |
| PHAGE_Bacill_G_NC_02 3719 (gi593777800) | - | + | - | - | - |
| Staphylococcal complement inhibitor (gi118725117) | + | + | + | + | + |
| chemotaxis-inhibiting protein CHIPS (gi588498304) | + | - | - | + | - |
| truncated amidase (gi30043930) | + | - | - | + | - |
| hypothetical | - | - | - | + | - |
| truncated amidase (gi118725115) | - | - | - | - | + |
| STAPHYLOKINASE PRECURSOR (gi30043932) | + | - | - | - | + |
| autolysin (gi526003579) | + | - | - | - | + |
| holin homolog (gi30043934) | + | - | - | - | - |
| hypothetical | - | + | - | - | - |
| transposase (gi9635740) | - | + | - | - | - |
| hypothetical protein (gi118725087) | - | + | - | - | - |
| hypothetical protein (gi239507391) | - | + | - | - | - |
| hypothetical protein (gi122891750) | - | + | - | - | - |
| dimeric dUTPase (gi509140128) | - | + | - | - | - |
| ORF114 (gi66395961) | - | + | - | - | - |
| hypothetical protein (gi509140130) | - | + | - | - | - |
| hypothetical protein (gi122891746) | - | + | - | - | - |
| hypothetical protein (gi215401132) | - | + | - | - | - |
| phi PVL orf 51-like protein (gi29028687) | - | + | - | - | - |
| dUTPase (gi971749902) | - | + | - | - | - |
| hypothetical | - | + | - | - | - |
| ORF008 (gi66395596) | - | + | + | - | - |
| holin (gi431810283) | - | + | + | - | - |
| holin homolog (gi30043934) | - | - | - | - | + |
| hypothetical protein (gi30043935) | + | + | + | - | + |
| enterotoxin P (gi30043936) | + | - | - | - | + |
| hypothetical protein (gi30043938) | + | - | - | - | + |
| hypothetical protein (gi30043939) | + | - | - | - | + |
| amidase (gi156604015) | - | - | - | + | - |
| holin (gi966198911) | - | - | - | + | - |
| phi PVL ORF 17 homologue (gi9635733) | - | - | - | + | - |
| phi PVL ORF 22 homologue (gi9635732) | - | - | - | + | - |
| hypothetical protein (gi30043940) | + | - | - | + | + |
| hypothetical protein (gi30043941) | + | - | - | + | + |
| 77ORF058 (gi41189563) | - | + | - | - | - |
| phi PVL ORF 17 homologue (gi9635733) | + | + | + | - | - |
| phi PVL ORF 22 homologue (gi9635732) | - | + | + | - | - |
| hypothetical protein (gi9635731) | - | - | + | - | - |
| 77ORF002 (gi41189517) | - | - | + | - | - |
| hypothetical protein (gi30043942) | + | - | + | + | + |
| hypothetical protein (gi118725108) | - | + | - | - | - |
| phage minor structural protein (gi118725107) | - | + | - | - | - |
| hypothetical protein (gi118725106) | - | + | - | - | - |
| phage tail tape measure protein (gi118725105) | - | + | - | - | - |
| tail length tape-measure protein (gi725916006) | - | - | + | - | - |
| hypothetical protein (gi30043943) | + | - | + | + | + |
| 77ORF100 (gi41189576) | + | + | + | + | - |
| hypothetical protein (gi118725103) | - | + | - | - | - |
| hypothetical protein (gi118725102) | - | + | - | - | - |
| tail superfamily protein (gi588498291) | - | + | - | - | - |
| hypothetical protein (gi118725100) | - | + | - | - | - |
| hypothetical protein (gi118725099) | - | + | - | - | - |
| head-tail adaptor (gi744692788) | - | + | - | - | - |
| hypothetical protein (gi118725096) | - | + | - | - | - |
| capsid protein (gi744692789) | - | + | - | - | - |
| putative Clp protease (gi118725094) | - | + | - | - | - |
| phage portal protein (gi118725093) | - | + | - | - | - |
| phage terminase (gi118725092) | - | + | - | - | - |
| hypothetical protein (gi118725091) | - | + | - | - | - |
| hypothetical protein (gi118725090) | - | + | - | - | - |
| hypothetical protein (gi118725089) | - | + | - | - | - |
| hypothetical protein (gi118725088) | - | + | - | - | - |
| hypothetical protein (gi725916005) | - | - | - | - | + |
| hypothetical protein (gi30043944) | + | - | + | + | + |
| hypothetical protein (gi744692809) | + | - | - | + | + |
| hypothetical protein (gi588498292) | - | - | + | - | - |
| hypothetical protein (gi30043945) | - | - | + | - | + |
| hypothetical protein (gi30043946) | + | - | + | + | + |
| hypothetical protein (gi30043947) | + | - | + | + | + |
| hypothetical protein (gi30043948) | + | - | + | + | + |
| hypothetical protein (gi30043949) | + | - | - | + | + |
| hypothetical protein (gi30043950) | + | - | + | + | + |
| hypothetical protein (gi30043951) | + | - | + | + | + |
| hypothetical protein, similar to scaffolding protein (gi30043952) | + | - | + | + | + |
| hypothetical protein (gi30043953) | + | - | + | + | + |
| hypothetical protein (gi30043954) | + | - | + | + | + |
| terminase (gi744692792) | - | - | - | - | + |
| hypothetical protein (gi30043955) | + | - | + | + | + |
| 77ORF040 (gi41189551) | + | - | - | + | - |
| HNH endonuclease (gi744692793) | - | - | + | - | + |
| hypothetical protein (gi30043957) | + | - | + | + | + |
| phi PVL ORF 60 homologue (gi9635710) | + | - | + | + | + |
| 77ORF019 (gi41189534) | + | - | - | + | - |
| hypothetical protein (gi30043959) | + | - | + | + | + |
| hypothetical protein (gi30043960) | + | - | + | + | - |
| hypothetical protein (gi744692815) | - | - | - | - | + |
| hypothetical protein (gi971755489) | - | - | - | - | + |
| ORF063 (gi66396316) | + | - | + | + | + |
| putative dUTP diphosphatase (gi526244901) | + | - | - | + | - |
| hypothetical protein (gi971750181) | + | - | - | - | - |
| hypothetical protein (gi971742272) | + | - | - | - | - |
| hypothetical protein (gi9635213) | + | + | - | - | - |
| hypothetical protein (gi526003559) | - | + | - | - | - |
| hypothetical protein (gi9635211) | - | + | - | - | - |
| ssDNA-binding protein (gi971749889) | - | + | - | - | - |
| hypothetical protein (gi118725074) | - | + | - | - | - |
| hypothetical protein (gi156603908) | + | - | - | + | - |
| hypothetical protein (gi30043962) | - | - | + | - | - |
| ORF090 (gi66395717) | - | - | + | - | - |
| ORF074 (gi66395713) | - | - | + | - | - |
| PVL phage protein (gi388570356) | - | + | + | - | - |
| dUTPase (gi744692795) | - | - | - | - | + |
| hypothetical protein (gi971755492) | - | - | - | - | + |
| hypothetical protein (gi971755493) | - | - | - | - | + |
| hypothetical protein (gi971750178) | - | - | - | - | + |
| hypothetical protein (gi744692816) | - | - | - | - | + |
| hypothetical protein (gi30043967) | - | - | + | + | + |
| hypothetical protein (gi30043968) | - | - | + | - | + |
| hypothetical protein (gi30043969) | + | - | + | + | - |
| phage regulatory protein (gi388570358) | - | - | - | - | + |
| single-strand DNA-binding protein (gi30043970) | + | - | + | + | + |
| hypothetical protein (gi526003558) | + | - | - | + | + |
| hypothetical protein (gi30043971) | - | - | + | - | - |
| RecT protein (gi388570318) | + | + | + | + | + |
| hypothetical protein (gi118725072) | - | + | - | - | - |
| RecF/RecN/SMC N terminal domain protein (gi388570319) | + | - | - | + | - |
| hypothetical protein (gi156603902) | + | - | - | - | - |
| hypothetical protein (gi30043973) | - | - | - | - | + |
| hypothetical protein (gi30043975) | + | - | - | + | + |
| hypothetical protein (gi971765387) | - | - | - | + | - |
| hypothetical protein (gi156603903) | - | - | + | - | - |
| hypothetical protein (gi156603901) | - | + | + | - | - |
| hypothetical protein (gi156603899) | + | - | + | + | - |
| hypothetical protein (gi30043976) | - | - | - | - | + |
| hypothetical protein (gi30043978) | - | - | - | - | + |
| hypothetical protein (gi30043979) | + | - | - | - | + |
| hypothetical protein (gi30043980) | + | - | - | - | + |
| ORF018 (gi66396131) | + | - | - | - | - |
| hypothetical protein (gi509140151) | - | - | + | + | - |
| hypothetical protein (gi118725066) | - | + | - | - | - |
| hypothetical protein (gi118725065) | - | + | - | - | - |
| hypothetical protein (gi118725064) | - | + | - | - | - |
| anti-repressor protein (gi526003584) | - | - | - | + | - |
| anti repressor (gi30043981) | - | - | - | - | + |
| hypothetical protein (gi30043982) | - | - | - | - | + |
| hypothetical transcriptional regulator (gi30043984) | - | - | - | + | + |
| similar to repressor (gi30043985) | - | - | - | + | + |
| anti-repressor (gi29028674) | - | + | + | - | - |
| hypothetical protein (gi156603895) | + | + | + | - | - |
| phi PVL orf 32-like protein (gi29028672) | + | + | + | - | - |
| Cro (gi9635683) | + | - | - | - | - |
| repressor (gi431810247) | + | - | - | - | - |
| hypothetical protein (gi557308086) | + | - | - | + | - |
| hypothetical protein (gi9635681) | + | - | - | - | - |
| hypothetical protein (gi509140158) | + | - | - | - | - |
| hypothetical protein (gi971742295) | + | - | - | - | - |
| hypothetical protein (gi557308083) | + | - | - | - | - |
| hypothetical protein (gi17426232) | - | - | - | + | - |
| ORF022 (gi66395530) | - | - | - | + | - |
| probable ATP-dependent helicase (gi30043986) | - | - | - | - | + |
| hypothetical protein (gi509139882) | - | - | - | - | + |
| hypothetical protein (gi30043987) | - | - | - | + | + |
| probable ss-1,3-N-acetylglucosaminyltransferase (gi30043988) | - | - | - | + | - |
| hypothetical protein (gi30043989) | - | - | - | + | - |
| hypothetical protein (gi257136358) | - | - | - | - | + |
| integrase (gi30043990) | + | - | - | - | - |
| integrase (gi509140091) | - | + | + | + | + |
| **attR (TGTATCCGAATTGG)** | + | + | + | - | - |
| **attL (AGTCTCCATATAGG)** | - | - | - | - | + |
| Hypothetical protein | + | + | + | + | - |
| Prohead core protein protease (gi100061) | - | - | - | + | - |
| **attL (TGAACCCAGTTTGG)** | - | - | - | + | - |
| LukF-PV(P83) precursor (gi9635737) | + | + | + | - | - |
| LukM precursor (gi9635736) | + | + | + | - | - |
