# Supplementary material for: Staphylococcus aureus ST398 Virulence Is Associated With Factors Carried on Prophage ϕSa3
Source: Front Microbiol. 2019 Sep 24;10:2219. doi: 10.3389/fmicb.2019.02219 (PMC6771273; doi:10.3389/fmicb.2019.02219)
Supplement: Supplementary file 2 [file Table_2.DOCX]

**Supplementary Table 2. Comparison of the *attL*/*attR* sites and the genes coding for proteins /hypothetical proteins among phage Group 2 (φSa3-G2) strains**

| ***attL*/*attR* sites and genes coding for proteins/hypothetical proteins** | **Strains** | | | | |
| --- | --- | --- | --- | --- | --- |
|  | **GD1108** | **293G** | **232N** | **387N** | **215N** |
| **attL (TGTATCCGAATTGG)** | + | + | + | + | + |
| hypothetical protein (gi588498306) | - | + | + | + | + |
| complement inhibitor SCIN (gi588498305) | + | + | + | + | + |
| chemotaxis-inhibiting protein CHIPS (gi588498304) | + | + | + | + | + |
| amidase (gi588498303) | + | + | + | + | + |
| hypothetical protein (gi588498302) | + | + | + | + | + |
| phi PVL ORF 17 homologue (gi9635733) | + | + | + | + | + |
| hypothetical protein (gi588498299) | + | + | + | + | + |
| hypothetical protein (gi588498298) | + | + | + | + | + |
| hypothetical protein (gi588498297) | + | + | + | + | + |
| tail protein (gi744692785) | + | + | + | + | + |
| tail tape measure protein (gi588498295) | + | + | + | + | + |
| hypothetical protein (gi588498294) | + | + | + | + | + |
| hypothetical protein (gi588498293) | + | + | + | + | + |
| hypothetical protein (gi588498292) | + | + | + | + | + |
| tail superfamily protein (gi588498291) | + | + | + | + | + |
| hypothetical protein (gi744692811) | + | + | + | + | + |
| hypothetical protein (gi588498289) | + | + | + | + | + |
| head-tail adaptor (gi588498288) | + | + | + | + | + |
| hypothetical protein (gi588498287) | + | + | - | + | + |
| hypothetical protein (gi588498286) | + | + | + | + | + |
| major capsid protein (gi588498285) | + | + | + | + | + |
| putative transposase (gi588498272) | + | - | - | - | - |
| putative Clp protease (gi588498284) | + | + | + | + | + |
| phage portal protein (gi118725093) | + | + | + | + | + |
| hypothetical protein (gi30043954) | + | + | + | + | + |
| hypothetical protein (gi588498280) | + | + | + | + | + |
| HNH endonuclease (gi588498279) | + | + | + | + | + |
| transcriptional activator (gi588498278) | + | + | + | + | + |
| phi PVL ORF 60 homologue (gi9635710) | + | + | + | + | + |
| 77ORF112 (gi41189581) | + | + | + | + | + |
| hypothetical protein (gi588498275) | + | + | + | + | + |
| hypothetical protein (gi588498274) | + | + | + | + | + |
| deoxyuridine 5'-triphosphate nucleotidohydrolase (gi725915984) | + | + | + | + | + |
| hypothetical protein (gi118725083) | + | + | + | + | + |
| hypothetical protein (gi588498271) | + | + | + | + | + |
| hypothetical protein (gi588498270) | + | + | + | + | + |
| hypothetical protein (gi588498269) | + | + | + | + | + |
| hypothetical protein (gi588498268) | - | + | + | + | + |
| endodeoxyribonuclease RusA (gi588498267) | + | + | + | + | + |
| hypothetical protein (gi588498266) | + | + | + | + | + |
| regulatory protein (gi588498265) | + | + | + | + | + |
| putative single-strand DNA-binding protein (gi588498264) | + | + | + | + | + |
| hypothetical protein (gi588498263) | + | + | + | + | + |
| RecT protein (gi588498262) | + | + | + | + | + |
| hypothetical protein (gi588498261) | + | + | + | + | + |
| hypothetical protein (gi588498259) | - | + | - | - | + |
| hypothetical protein (gi588498258) | + | + | + | + | + |
| hypothetical protein (gi122891726) | + | - | + | + | - |
| hypothetical protein (gi588498255) | + | + | + | + | + |
| hypothetical protein (gi588498254) | + | + | + | + | + |
| hypothetical protein (gi588498253) | + | + | + | + | + |
| hypothetical protein (gi588498252) | + | + | + | + | + |
| putative antirepressor (gi588498251) | + | + | + | + | + |
| hypothetical protein (gi588498250) | + | + | + | + | + |
| DNA-binding protein (gi588498249) | + | + | + | + | + |
| putative repressor (gi588498248) | + | + | + | + | + |
| putative exonuclease (gi588498247) | + | + | + | + | + |
| hypothetical protein (gi588498246) | - | + | - | - | + |
| hypothetical protein (gi588498245) | + | + | + | + | + |
| putative lipoprotein (gi588498244) | + | + | + | + | + |
| integrase (gi588498243) | + | + | + | + | + |
| **attR (TGTATCCAAACTGG)** | + | + | + | + | + |
| hypothetical protein | + | + | + | + | + |
| LukF-PV(P83) precursor (gi9635737) | + | + | + | + | + |
| LukM precursor (gi9635736) | + | + | + | + | + |
